# Predictors of haematocrit in lumbar fusion for lumbar disc herniation: a surgical assessment

**DOI:** 10.1186/s12891-017-1655-5

**Published:** 2017-08-01

**Authors:** Qingchun Cai, Sixiang Zeng, Liqiang Zhi, Junlong Wu, Wei Ma

**Affiliations:** Department of Orthopedics, First Affiliated Hospital of Medical College, Xi’an Jiaotong University, 277 Yanta West Street, Xi’an, 710061 Shaanxi People’s Republic of China

**Keywords:** Haematocrit, Posterior lumbar fusion, Surgical outcome, Haemorrhage

## Abstract

**Background:**

Low haematocrit (Hct) is associated with a higher rate of post-operative complications, increased mortality, and additional medical costs following cardiac surgery. Predictors of post-operative Hct in lumbar fusion are unclear and may be beneficial in avoiding adverse surgical outcomes.

**Methods:**

A total of 704 lumbar disc herniation patients (385 males, 319 females) who underwent primary lumbar fusion surgery were reviewed in this retrospective study.

**Results:**

In the 687 patients who met the selection criteria, the pre-operative Hct was 41.23 ± 4.57%, the post-operative Hct was 32.61 ± 4.52%, the peri-operative Hct decline was 8.62 ± 4.07%, the estimated intra-operative blood loss was 586.76 ± 346.62 mL, and the post-operative drainage was 489.33 ± 274.32 mL. Pre-operative Hct, estimated blood volume, estimated intra-operative blood loss, post-operative drainage, allogeneic blood transfusion, and age showed significant correlations with post-operative Hct, and all factors were involved in the final multiple regression model. Patients who received intensive care had lower post-operative Hct values, and the length of post-operative hospital stay was negatively correlated with post-operative Hct.

**Conclusions:**

Dangerously low post-operative Hct is related to the length of ICU stay and post-operative hospital stay. Age, pre-operative Hct, intra-operative blood loss, post-operative drainage, and units of allogeneic blood transfusion are significant predictors of post-operative Hct and Hct decline. Hct variations during the operation make the calculation of total blood loss difficult.

## Background

Lumbar fusion rates have risen rapidly since the 1980s, a tendency driven by the approval of new surgery implants, enhanced diagnostic awareness, and a change in patient demand [1–5]. Posterior lumbar fusion is an effective therapy for primary lumbar disc herniation in patients suffering from chronic back pain or patients who suffer from concomitant canal stenosis, instability associated with radiculopathy, extreme lateral prolapse of a lumbar intervertebral disc, or multiple disc herniation; this procedure is conventionally performed at many medical institutions [6, 7]. Several published studies have indicated that the volume of peri-operative blood loss during posterior lumbar fusion fluctuates across a wide range [8–12]; massive blood loss during cardiac surgery causes lower post-operative Hct, which is associated with a higher rate of post-operative complications, increased mortality, and additional medical expenditures [13–18]. When massive haemorrhage occurs during surgery, crystalloid and colloidal solutions are applied to maintain intravascular volume and improve microcirculation; furthermore, blood products are necessary to enhance the ability to carry oxygen and improve the blood coagulation function if haemorrhage is unremitting. However, standard liquid management and allogeneic blood transfusion have the opposite effect on Hct, which hinders the prediction of post-operative Hct. The Hct level is a crucial variable for the calculation of total blood loss. Therefore, this study attempted to answer several questions: 1) Is a lower Hct level associated with poor surgical outcomes in lumbar fusion? 2) What are the dominant predictors of post-operative Hct? 3) Do variations in Hct affect the routine method for calculating total blood loss?

## Methods

### Case sourcing and selection

The cohort in this retrospective study included patients who had undergone posterior lumbar fusion because of lumbar disc herniation between June 2009 and September 2014. The inclusion criteria were as follows: a diagnosis of lumbar disc herniation or in parallel with lumbar canal stenosis, a primary surgical procedure combined with decompression, discectomy, intervertebral or intertransverse lumbar fusion, and internal fixation with a pedicle screw performed under general anaesthesia. The exclusion criteria were as follows: revision surgery, clotting abnormalities or anticoagulant use in the week prior to surgery, use of blood conservation strategies, multiple incisions, and leakage of cerebrospinal fluid intra-operatively or post-operatively.

### Collection of variables

Demographic data from the patients were collected as follows: month of admission, age, sex, contact details, height and weight. The hospital and surgical data collected included the following: smoking history, alcohol history, body mass index (BMI), liver disease, anaemia, Charlson Comorbidity Index (CCI) [19], ASA physical status classes index (ASA), National Nosocomial Infections Surveillance (NNIS) (pre-assessment scale), surgery segments, surgeon-estimated volume of intra-operative blood loss, anaesthetist-estimated volume of intra-operative blood loss, units of allogeneic blood transfusion (units of ABT), pre-operative Hct (Hct[Pre]), pre-operative haemoglobin (Hb[pre]), post-operative Hct (Hct[Post]), post-operative haemoglobin (Hb[post]), post-operative drainage (PD), length of post-operative hospital stay (Length of Post-op Hos stay), length of ICU stay, occurrence of redo surgery, wound infection, urinary infection, pneumonia, sepsis, myocardial infarction, deep vein thrombosis, pulmonary embolism, and post-operative death. The transfusion triggers at the authors’ institution were as follows: 1) haemoglobin level less than 9 g/dL; 2) symptomatic anaemia (shortness of breath, fatigue, dizziness, decreased exercise tolerance, and so on); and 3) a liberal transfusion trigger adopted when inadequate oxygenation would exacerbate the patient’s pre-existing complication [20, 21]. The principle of liquid therapy at the institution conformed to the standards in the review by Sweeney [22] and the latest intravenous liquid therapy guidelines developed by the National Clinical Guideline Center [23]. A routine surgical procedure was performed by several senior spinal surgeons for all of the reviewed patients, and the application of intervertebral lumbar fusion or intertransverse lumbar fusion was performed according to their judgement. The surgeon and anaesthetist separately estimated the intra-operative blood loss through weighing sponges, suction drainage and errhysis from the surgical field. Negative pressure drainage was used for all patients to prevent post-operative haematoma and associated neurologic compromise [24], and the drainage was removed 2 or 3 days after surgery. The haemoglobin and Hct data were derived from routine pre-operative and post-operative blood tests. The blood samples were prepared by nurses with an EDTAK blood anticoagulant tube before surgery and after the drainage was removed. The blood samples were immediately delivered to the laboratory and analysed with a Sysmex HST302 or Sysmex HST201 automatic blood cell analysis assembly line.

### Calculation of blood loss

The average of volume of intra-operative blood loss estimated by the surgeon and by the anaesthetist was calculated as estimated intra-operative blood loss (EBL[intra]).

The calculation of blood volume was based on the classic method developed by Nadler and colleagues [25]:

EBV = K1*height (m)^3^ + K2*weight (kg) + K3.

[K1 = 0.3669, K2 = 0.03219, K3 = 0.6041 for males and K1 = 0.3561, K2 = 0.03308, and K3 = 0.1833 for females].

To maintain dimensional consistency, the EBV was converted to the estimated blood volume in millilitres (EBV[mL]): EBV(mL) = EBV*1000.

The Hct decline was calculated as follows:

Hct Decline = Hct (pre)-Hct (post).

[Hct Decline is the scale of Hct decline during peri-operation, Hct (pre) is pre-operative Hct, and Hct (post) is post-operative Hct].

### Statistical analysis

The statistical analysis was performed with IBM SPSS statistics software version 19 (IBM Co, Armonk, NY, USA). The measurement data are shown as the mean ± standard deviation, and the qualitative and ordinal data were demonstrated as frequency distributions. The independent sample t-test was applied to compare post-operative Hct changes between two different groups. One-way ANOVA analysis was applied to compare post-operative Hct changes among three or more groups. If the test of homogeneity of variance was inappropriate, the Kruskal-Wallis test was applied for multiple independent samples of non-parametric tests. Pearson’s correlation coefficient was used to explore the correlation between continuous variables. Multiple linear regression was used to explore the predictors of post-operative Hct with a stepwise regression strategy while controlling for smoking, alcohol drinking, anaemia and NNIS. Throughout data analysis, *P* < 0.05 was considered statistically significant.

## Results

A total of 687 lumbar disc herniation patients (375 males, 312 females) of the 704 reviewed patients met the selection criteria. The statistical descriptions of the demographic data, hospital data, and surgery data are provided in Tables 1 and 2. The EBV(mL) was 4268.57 ± 665.11 mL, the EBL(intra) was 586.76 ± 346.62 mL, the volume of PD was 489.33 ± 274.32 mL, and the units of ABT were 1.49. Most patients (68.0%) scored two points on the ASA physical status classes index assessment. A total of 342 patients (49.8%) underwent fusion surgery of a single segment, 292 patients (42.5%) underwent fusion surgery of two segments, and the remaining 53 patients (7.7%) underwent fusion surgery of three or four segments.

**Table 1:** Description of quantitative data

	Minim	Maximum	Mean	Std-deviation
Age (Y)	18	80	52.86	12.38
Height (cm)	1.45	1.86	1.67	0.07
Weight (Kg)	40	126	66.40	11.16
BMI index (Kg/m^2^)	15.63	38.89	23.78	3.25
BL(surgeon) (mL)	50	2800	574.35	337.81
BL(anaesthetist) (mL)	50	2800	599.17	375.67
EBL(intra) (mL)	75	2800	586.76	346.62
PD (mL)	50	2065	489.33	274.32
Units of ABT (U)	0	11	1.49	1.84
Hb(pre) (g/L)	85	188	137.74	16.79
Hct(pre) (%)	28.7	72.4	41.23	4.57
Hb(post) (g/L)	72	164	109.2	16.14
Hct (post) (%)	21.3	46.7	32.61	4.52
Hct Decline(%)	−4.2	35.6	8.62	4.07
EBV(mL)	2625.20	6799.80	4268.57	665.11
Length of Post-op Hos Stay (Day)	3	86	12.4	5.8

*BL (surgeon)* volume of intra-operative blood loss estimated by the surgeon, *BL (anaesthetist)* volume of intra-operative blood loss estimated by the anaesthetist, *EBL (intra)* estimated intra-operative blood loss, *PD* post-operative drainage, *Units of ABT* units of allogenic red blood cell transfusion, *Hb (pre)* pre-operative haemoglobin, *Hct (pre)* pre-operative haematocrit, *Hb (post)* post-operative haemoglobin, *Hct (post)* post-operative haematocrit, *Hct decline* decline of haematocrit, *EBV(mL)* estimated blood volume in millilitres, *Length of Post-op Hos Stay* The length of the post-operative hospital stay

**Table 2:** Post-operative haematocrit and haematocrit decline among different groups

		Frequency	Hct(post) (%)	*P* value	Hct decline (%)	*P* value
Gender	Male	375(54.6%)	34.54 ± 4.32	<0.001	9.20	<0.001
	Female	312(45.4%)	30.30 ± 3.58		7.92	
Smoking	Yes	189(27.5%)	34.70 ± 4.21	<0.001	8.85	.365
	No	498(72.5%)	31.82 ± 4.38		8.53	
Alcohol Drinking	Yes	43(6.3%)	35.31 ± 4.29	<0.001	8.91	.631
	No	644(93.7%)	32.43 ± 4.48		8.60	
Liver Disease	Yes	12(1.7%)	32.28 ± 3.96	.799	8.86	.837
	No	675(98.3%)	32.62 ± 4.53		8.61	
Anaemia	Yes	32(4.7%)	27.86 ± 2.46	<0.001	4.95	<0.001
	No	655(95.3%)	32.85 ± 4.47		8.80	
CCI	0	462(67.2%)	32.88 ± 4.47	.103	8.51	.684
	1	162(23.6%)	31.86 ± 4.46		8.95	
	2	53(7.7%)	32.91 ± 5.08		8.49	
	3	9(1.3%)	30.88 ± 3.92		9.18	
	5	1(0.1%)	31.70		9.80	
ASA	1	137(19.9%)	33.42 ± 4.69	.018	8.38	.217
	2	467(68.0%)	32.46 ± 4.43		8.70	
	3	79(11.5%)	31.90 ± 4.55		8.73	
	4	4(0.6%)	36.70 ± 3.70		5.1	
NNIS	0	516(75.1%)	32.81 ± 4.55	.096	8.40	.043
	1	162(23.6%)	31.95 ± 4.38		9.44	
	2	9(1.3%)	33.19 ± 4.74		6.29	
Surgery Segments	1	342(49.8%)	33.26 ± 4.43	<0.001	7.94	<0.001
	2	292(42.5%)	32.16 ± 4.54		9.10	
	3	48(7.0%)	30.78 ± 4.45		10.70	
	4	5(0.7%)	32.48 ± 2.65		7.04	
Length of ICU Stay	YES	67(9.8%)	31.42 ± 4.06	0.022	8.93	0.507
	NO	620(90.2%)	32.74 ± 4.55		8.58	
Occurrence of Redo Surgery	YES	11(1.6%)	33.36 ± 4.99	0.580	5.64	0.014
	NO	676(98.4%)	32.60 ± 4.52		8.67	
Wound Infection	YES	26(3.8%)	31.13 ± 4.12	0.089	8.09	0.499
	NO	661(96.2%)	32.67 ± 4.53		8.64	

*Hct (post)* post-operative haematocrit, *HCT decline* decline of haematocrit, *CCI* Charlson Comorbidity Index, *ASA* American Society of Anaesthesiologists Physical Status Classes Index, *NNIS* National Nosocomial Infections Surveillance (pre-assessment scale)

### Post-operative Hct

The HCT(pre) was 41.23 ± 4.57%, and the HCT(post) was 32.61 ± 4.52%. The post-operative Hct of the male subjects was 34.54 ± 4.32%, which was higher than that of the females (*p* < 0.001), and patients with a history of smoking, alcohol abuse or anaemia had lower post-operative Hct (*p* < 0.001). The post-operative Hct change decreased significantly as the fusion segments increased (Table 2). Age, BMI, EBV(mL), Hct(pre), EBL(intra) and PD showed statistical correlations with post-operative Hct in the Pearson correlation analysis (Table 3). Six factors were involved in the ultimate multiple regression model of post-operative Hct (in accordance with the standard coefficient): Hct(pre), BL(intra), units of ABT, PD, EBV(mL), and age. This model explained 53% of the post-operative Hct (R^2^ = 0.530) (Table 4).

**Table 3:** Pearson correlation analysis about post-operative haematocrit, haematocrit decline and average haematocrit

	Hct(post)		Hct decline		Hct(average)	
	Correlation	*P* value	Correlation	*P* value	Correlation	*P* value
Age	−0.232	<0.001	0.112	.003	−0.202	<0.001
BMI Index	0.130	.001	0.032	.400	0.161	<0.001
EBV(mL)	0.455	<0.001	0.127	.001	0.569	<0.001
Hct(pre)	0.600	<0.001	0.457	<0.001	0.896	<0.001
EBL(intra)	−0.219	<0.001	0.224	<0.001	−0.131	.001
PD	−0.240	<0.001	0.271	<0.001	−0.131	.001
Length of Post-op Hos Stay	−0.106	.005	−0.013	0.730	−0.125	0.001

*Hct (post)* post-operative haematocrit, *Hct (average)* the average of pre-operative haematocrit and post-operative haematocrit, *Hct decline* decline of haematocrit, *EBV (mL)* estimated blood volume in millilitres, *Hct (pre)* pre-operative haematocrit, *EBL (intra)* estimated intra-operative blood loss, *PD* post-operative drainage, *Length of Post-op Hos Stay* The length of the post-operative hospital stay

**Table 4:** Multiple regression analysis model about post-operation haematocrit and haematocrit decline

		Unstandardized coefficient	Standardized coefficient	VIF	R square
Hct (post)	Constant	11.181			0.53
	Hct(pre)	0.504	0.509	1.512	
	PD	−0.004	−0.231	1.266	
	EBV(mL)	0.001	0.213	1.484	
	EBL(intra)	−0.006	−0.434	2.573	
	Units of ABT	1.016	0.414	2.683	
	Age	−0.035	−0.096	1.126	
Hct Decline	Constant	−13.690			0.42
	Hct(pre)	0.513	0.576	1.702	
	PD	0.004	0.256	1.266	
	Gender	−1.760	−0.216	1.602	
	EBL(intra)	0.006	0.482	2.642	
	Units of ABT	−0.996	−0.451	2.702	
	BMI index	−0.153	−0.123	1.080	
	Age	0.040	0.121	1.138	

*VIF* variance inflation factor, *Hct (post)* post-operative haematocrit, *Hct decline* decline of haematocrit, *Hct (pre)* pre-operative haematocrit, *PD* post-operative drainage, *EBV (mL)* estimated blood volume in millilitres, *EBL (intra)* estimated intra-operative blood loss, *Units of ABT* units of allogenic red blood cell transfusion

### Hct decline

The peri-operative Hct decline was 8.62 ± 4.07%. The extent of the decline in peri-operative Hct was significantly different according to gender and surgery segments. Anaemia caused a smaller decline in Hct (Table 2). The Pearson correlation analysis revealed that the Hct decline was positively correlated with age, EBV(mL), Hct (pre), EBL (intra), and PD (Table 3). A multiple regression equation for Hct decline was established, and the equation included the following predictors: Hct (pre), PD, gender, EBL (intra), units of ABT, BMI, and age (Table 4).

### Average Hct

Average Hct was defined as the average between the pre-operative Hct and post-operative Hct. The average Hct also had a negative correlation with EBL(intra) and PD, indicating that more peri-operative blood loss was associated with a lower average Hct. Age, BMI, and EBV(mL) were also statistically correlated with average Hct (Table 3).

### Surgical outcomes

The length of post-operative hospital stay was 12.4 ± 5.8 days; 67 patients (9.8%) received intensive care, and the mean length of ICU stay was 2.5 ± 1.0 days. A total of 11 patients (1.6%) underwent redo surgeries, 26 patients (3.8%) suffered from wound infection, and 5 (0.7%) and 3 (0.4%) patients encountered urinary infection and pneumonia, respectively. One patient suffered from deep vein thrombosis, but no patients experienced sepsis, pulmonary embolism or post-operative death. Patients who received intensive care had lower post-operative Hct; patients who underwent redo surgeries had smaller Hct declines; and the length of post-operative hospital stay had a negative correlation with post-operative Hct and average Hct (Tables 2 and 3).

## Discussion

The intra-operative blood loss was 586.76 ± 346.62 mL, which was less than the 907 ± 775 mL estimated blood loss during posterior spinal fusion for adolescent idiopathic scoliosis [11]. Several published studies have confirmed that an increased number of fusion segments increases the volume of intra-operative blood loss [10, 12, 26–28]. In this study, 92.3% of patients underwent a one or two segment fusion procedure, whereas only five patients underwent the highest four segment fusion procedure. Thus, fewer fusion segments explained the lower intra-operative blood loss. The volume of post-operative drainage was larger than that reported in a study performed by Zou, which involved one-segment or two-segment lumbar spinal surgery [29]. In the present study, the negative pressure type drainage influenced the volume of post-operative drainage; furthermore, not all patients underwent lumbar fusion procedures in Zou’s study. Taken together, these data suggest that more surgery segments led to more peri-operative blood loss.

To measure the effect of these collected variables on post-operative Hct, a multiple regression analysis was performed. The multiple regression model of post-operative Hct showed the following: 1) pre-operative Hct had the most predictive effect on post-operative Hct in this model; 2) post-operative drainage and intra-operative blood loss were important predictors for post-operative Hct on the basis of standardised coefficients; 3) a prudent transfusion strategy and elderly resulted in lower post-operative Hct. Lee reported that post-operative Hct was not correlated with age, BMI, or anaesthesia type [30]. However, Wang and Hannan suggested that age may be associated with changes in peri-operative Hct [13, 31]. With a stepwise strategy, age was entered in the multiple regression model in this study but had a very weak prediction effect. Additionally, females had lower post-operative Hct, although the extent of Hct decline was less obvious mathematically because females had lower pre-operative Hct.

Increased haemodilution is associated with vital organ dysfunction, other peri-operative complications, an increase in post-operative mortality, and greater consumption of medical resources in cardiac and non-cardiac surgery [13–18]. A published animal experiment study also confirmed that a lower Hct level was associated with lower perfusion pressure, increased fluid retention, and worse cerebrovascular injury after deep hypothermic circulatory arrest [32]. In the present study, patients who received intensive care had lower post-operative Hct; the length of post-operative hospital stay was negatively correlated with post-operative Hct and average Hct. These data indicated that patients with lower post-operative Hct required more medical expenditure, which is consistent with results from previous studies. Interestingly, patients who underwent redo surgeries had a lesser Hct decline. After a comprehensive review of these cases, 8 of the 11 patients underwent redo surgeries for symptomatic epidural haematoma, which signified that the post-operative drainage was insufficient and caused a lesser Hct decline. Considering the low incidence of urinary infection, pneumonia, sepsis, deep vein thrombosis, pulmonary embolism and post-operative death in lumbar fusion, larger samples are needed to explore the relationship between these events and post-operative Hct.

Both the Pearson correlation analysis and multiple regression analysis regarding Hct decline showed that age, Hct(pre), EBL(intra) and PD were positively correlated with Hct decline. It is evident that Hct declines to a greater extent when more blood is lost during surgery. However, the positive correlation between Hct decline and Hct(pre) is puzzling. Considering that there is no consistent blood transfusion threshold, a lower chance of receiving blood transfusion peri-operatively may contribute to a greater decline in Hct among patients with a higher pre-operative Hct. The negative standardised coefficient of units of ABT in the Hct decline multiple regression model also confirmed this conjecture from another aspect. This means that appropriate blood transfusion is very important for avoiding lower Hct. Therefore, a liberal transfusion strategy should be applied to patients who undergo lumbar fusion with three or more segments to avoid lower post-operative Hct due to the relationship between post-operative Hct and the length of post-operative hospital stay/ICU stay. Yoshihara and Jans found that pre-operative anaemia was a significant predictor of allogeneic blood transfusion in hip and knee arthroplasty [33, 34]. Patients diagnosed with anaemia had less of a decline in peri-operative Hct, perhaps because of the change in blood transfusion strategy.

Following the method developed by Gross et al., many published studies have calculated total blood loss as follows: total blood loss = estimated blood volume*(pre-operative Hct - post-operative Hct)/the average Hct [35–39]. This formula calculates the volume of red blood cell loss according to pre-operative Hct and post-operative Hct and then converts the volume of red blood cell loss into the volume of blood loss using the average Hct. As the Pearson correlation analysis of average Hct showed, greater peri-operative haemorrhage causes a lower average Hct level (see details in Table 3). Based on the aforementioned formula, the volume of blood loss is converted by lower average Hct when more haemorrhage occurs, which means that the same calculated volume of blood loss would contain different volumes of red blood cells due to different average Hct. A differential red blood cell proportion indicates a different oxygen delivery ability. The other problem is that this formula was originally used to estimate allowable pre-transfusion blood loss, so only the influences of blood loss and fluid management on the fluctuation of peri-operation Hct were considered. In such a simplified mathematical model, the Hct declines linearly during surgery. However, the effects of allogeneic transfusion and blood conservation strategies on Hct, which could affect the accuracy of the blood loss calculation, were ignored.

## Conclusion

Lower post-operative Hct is correlated with ICU stay and the length of post-operative hospital stay. Age, pre-operative Hct, intra-operative blood loss, post-operative drainage, and units of allogeneic blood transfusion are significant predictors of post-operative Hct and Hct decline. Adoption of a liberal transfusion strategy in further studies is needed to enforce guidelines for its use in lumbar fusion surgeries with multiple segments. The fluctuation in Hct is influenced by the volume of blood loss, liquid therapy, blood product transfusion, and other blood conservation strategies that make the routine calculation method for total blood loss inaccurate.

## Abbreviations

ABT [intra]: Volume of intra-operative allogeneic blood transfusion
ABT[post]: Post-operative allogeneic blood transfusion
ASA: ASA physical status classes index
BL[anaesthetist]: Volume of intra-operative blood loss estimated by the anaesthetist
BL[surgeon]: Volume of intra-operative blood loss estimated by the surgeon
BMI index: Body mass index
CCI: Charlson Comorbidity Index
EBL (intra): Estimated intra-operative blood loss
EBV (ml): Estimated blood volume in millilitres
EBV: Estimated blood volume
Hb [post]: Post-operative haemoglobin
Hb [pre]: Pre-operative haemoglobin
Hct [Pre]: Pre-operative haematocrit
Hct decline: Decline of haematocrit
Hct[Post]: Post-operative haematocrit
Length of Post-op Hos Stay: The length of the post-operative hospital stay
NNIS: National Nosocomial Infections Surveillance
PD: Post-operative drainage
units of ABT: The sum of ABT (intra) and ABT (post)
